# High endothelial venules predict response to PD-1 inhibitors combined with anti-angiogenesis therapy in NSCLC

**DOI:** 10.1038/s41598-023-43122-w

**Published:** 2023-09-30

**Authors:** Dafu Ye, Yao Jin, Yiming Weng, Xue Cui, Jinsong Wang, Min Peng, Qibin Song

**Affiliations:** Department of Oncology, Renmin Hospital of Wuhan University, Wuhan, Hubei People’s Republic of China

**Keywords:** Cancer, Biomarkers

## Abstract

Tumor-associated high endothelial venules (TA-HEVs) mediate lymphocyte entry into tumors. Therefore, combined anti-angiogenesis therapy and programmed death-1 (PD-1) inhibitors might stimulate tumor immunity. This study will explore the TA-HEVs and real-world data of the combination therapy in non-small cell lung cancer (NSCLC). Firstly, we found a certain relationship between HEVs and immune effector cells by multiple immunofluorescence staining. We then analyzed the efficacy of immunotherapy combined with anti-angiogenesis therapy in advanced NSCLC patients by collecting real-world clinical data. Finally, we explored the predictive value of HEVs in combination therapy by analyzing pre-treatment pathological slides of patients with multiple immunofluorescence and RNA sequencing. Immunofluorescence staining of high endothelial venules (PNAd+) reveals that the frequency of HEVs is positively correlated with tumor-infiltrating stem-like CD8+ T cells (TCF-1+PD-1+) in the TME of advanced NSCLC patients (P = 0.0221). We retrospectively analyzed the efficacy of 96 patients with advanced NSCLC who received PD-1 inhibitors combined with anti-angiogenesis therapy in the real-world. The median PFS of patients combined with anti-angiogenesis therapy was longer than that of patients without anti-angiogenesis therapy (9.7 vs 8.6 months, P = 0.041). Multiple immunofluorescence staining of tumor biopsies before treatment from 14 patients with advanced NSCLC reveals that PNAd+ is predictive of better response and survival upon PD-1 inhibitors combined with anti-angiogenesis therapy (P = 0.0274). In addition, we collected peripheral blood from an effective group of patients for RNA sequencing and found that immune cells activation-related gene expression scores were higher. Combined anti-angiogenic and anti-PD-1 therapy stimulates tumor immunity through TA-HEVs formation. TA-HEVs not only mediate immune cell entry into tumors, but also are associated with the efficacy of PD-1 inhibitors and anti-angiogenesis therapy in NSCLC.

## Introduction

Tumor-associated high endothelial venules (TA-HEVs) derive from post-capillary venules, express high levels of the high endothelial venule-specifc sulfated MECA-79 (PNAd) antigens^1,2^. TA-HEVs play an essential role in lymphocyte recirculation and the formation of tertiary lymphoid structures (TLSs)^3–5^. Recent studies have revealed immune checkpoint inhibitors (ICIs) therapy also increasing TA-HEVs and improving the infifiltration of CD8+ T cells^6–8^. Furthermore, anti-angiogenic therapy can also downregulate continuous angiogenic signaling and result in vasculature normalization which promoting the formation of TA-HEVs^9,10^. Despite these important advances, TA-HEVs remain poorly defined in immunotherapy of non-small cell lung cancer (NSCLC). Here, we will further explore the correlation between TA-HEVs and the efficacy of immunotherapy combined with anti-angiogenesis therapy in NSCLC.

## Methods

The study in this paper involves two parts: retrospective clinical study and basic research, which are mainly divided into the experimental steps in supplementary materials. For details, please refer to the text below.

### Analysis of the real world data

#### Study population

Advanced NSCLC patients admitted to the Cancer Center of Renmin Hospital of Wuhan University from December 2019 to January 2021 were collected. The enrolled patients met the following inclusion and exclusion criteria: (1) Histopathologically confirmed diagnosis of NSCLC; (2) the TNM stages were IIIB—IV; (3) the treatment regimen must include programmed cell death 1 (PD-1) inhibitors, with or without anti-angiogenic drugs; (4) all patients receive at least two courses of combination therapy (21 days as a course). Patients were followed up by telephone or outpatient, with the last follow-up in July 2021. All the clinical data collected above were retrospective.

#### Study variable

Objective response rate (ORR), disease control rate (DCR), progression-free survival (PFS) and safety were assessed.

PFS is defined as the time from receiving ICIs combined with or without anti-angiogenesis to disease progression or death of the patient. Common Terminology Criteria for Adverse Events (CTCAE) Version 5.0^11^ was used to assess patients’ adverse events (AEs). The expression level of programmed cell death ligand 1 (PD-L1) of tumor cells was scored using the tumor proportion score (TPS).

### Multiple immunofluorescence staining

#### Patient samples

A total of 14 pathological sections of patients in the treatment group before treatment were collected from the Pathological Center of Renmin Hospital of Wuhan University. All patients provided informed consent for sampling of their tissue. Formalin-fixed paraffin-embedded (FFPE) blocks were retrieved and 4 µm-thick slides were taken for multiple immunofluorescence staining^12^. Then the slices were divided into effective group (PFS > 6 months) and void group (PFS ≤ 6 months) according to PFS. In addition, we collected pre-treatment pathological sections from 42 patients with NSCLC who received subsequent immunotherapy with or without anti-angiogenesis therapy.

This study was approved by the Ethics Committee of Renmin Hospital of Wuhan University, Ethics No: WDRY2022-K041.

#### Multiplexed immunofuorescence

Formalin-fixed paraffin-embedded samples of primary tumors were immunostained using Opal™ reagents The impact of different reagent concentrations and quantities were analyzed with respect to signal specificity, stripping efficiency, staining order effects, and spectral signal isolation. Images were acquired on a Vectra 3.0® or Vectra Polaris automated imaging system, and analyzed with inForm® software^12^.

#### Image analysis

Fluorescent slides were scanned using the Vectra Polaris 3.0 (Akoya Biosciences, Marlborough, MA, United States of America) using 40 × magnifcation (Plan APO 40 × /NA 0.75, 0.25um/pixel) and auto-estimated exposure times. Whole slide scan was imaged using 5 epi-fuorescent flters (DAPI, Opal 480, Cy3, Cy5 and Opal 780). Individual TMA cores were selected using the TMA array in the Phenochart sofware for image acquisition and acquired with auto-estimated exposure times for each epi-fuorescent flter. Te full Opal 9 acquisition protocol requires use of 7 epi-fuorescent flters (DAPI, Opal 480, FITC, Cy3, Texas Red, Cy5 and Opal 780) imaging at 20 nm spectral bands as designed for the Vectra Polaris. Multiplex auto-fuorescent slide with no primary antibodies was created and scanned using the same exposure times as labelled multiplex slides. We then acquired image on an Akoya Vectra Polaris and performed multispectral unmixing using INFORM^13,14^.

### RNA sequencing

#### Patient cohort(s) and sample collection

A total of 14 patients who received immunotherapy combined with antiangiogenesis therapy were selected, then these patients were divided into effective group (PFS > 6 months, n = 7) and void group (PFS ≤ 6 months, n = 7) according to PFS. Collect blood samples of these patients before treatment, extract RNA from leukocytes after peripheral blood extraction, perform RNA sequencing.

#### RNA-seq data processing and quality check

RNA-seq FASTQ files were first processed through FastQC (v.0.11.5)45, a quality control tool to evaluate the quality of sequencing reads at both the base and read levels. The reads that had ≥ 15 contiguous lowquality bases (phred score < 20) were removed from the FASTQ files. STAR 2-pass alignment (v.2.5.3)46 was then performed on the filtered FASTQ files with default parameters to generate RNA-seq BAM file for each sequencing event. After that, RNA-SeQC (v.1.1.8)47 was run on the aligned BAM files to generate a series of RNA-seq related quality control metrics including read counts, coverage, and correlation. A matrix of Spearman correlation coefficients was subsequently generated by RNASeQC among all sequencing events. The correlation matrix was carefully reviewed and the sequencing event generated from one library pool^13,15^.

#### Deconvolution of the cellular composition with MCP-counter

The R package software MCP-counter18 was applied to the normalized log2-transformed FPKM expression matrix to produce the absolute abundance scores for major immune cell types CD3+ T cells, CD8+ T cells, cytotoxic lymphocytes, natural killer cells, B lymphocytes, monocytic lineage cells and myeloid dendritic cells^12^. The deconvolution profiles were then hierarchically clustered and compared across response and treatment groups^13^.

#### Gene signatures for the functional orientation

The signatures were the following^16,17^. T cells: CD28, CD3D, CD3G, CD5, CD6, CHRM3-AS2, CTLA4, FLT3LG, ICOS, MAL, PBX4, SIRPG, THEMIS, TNFRSF25 and TRAT1; CD8+ T cells: CD8B, cytotoxic lymphocytes: CD8A, EOMES, FGFBP2, GNLY, KLRC3, KLRC4 and KLRD1; B lineage: BANK1, CD19, CD22, CD79A, CR2, FCRL2, IGKC, MS4A1 and PAX5; Monocytic lineage: ADAP2, CSF1R, FPR3, KYNU, PLA2G7, RASSF4 and TFEC; Myeloid dendritic cells: CD1A, CD1B, CD1E, CLEC10A, CLIC2 and WFDC21P; T cell activation (CXCL9, CXCL10, CXCL16, IFNG and IL15), T cell survival (CD70 and CD27)^13,18^.

### Statistical analysis

Statistical analysis was performed using R version 3.3.3. Pearson chi-square test or Fisher's exact test (when there are less than 5 expected counts in the contingency table) is used to analyze the relationship between treatment response and clinical characteristics, and whether there are significant differences in baseline characteristics between the treatment group and the control group. Kaplan–meier method was used to analyze PFS, and the Log-rank test to compare the difference in survival outcomes between different groups. Then we used multivariate Cox regression to analyze the prognostic value of each clinical factor for patients with PFS. P < 0.05 was considered statistically significant.

### Ethics approval and consent to participate

This study was approved by the Ethics Committee of Renmin Hospital of Wuhan University, Ethics No: WDRY2022-K041. All methods were performed in accordance with the relevant guidelines and regulations.

## Results

### Correlation between HEVs, TLSs and stem-like CD8+ T cells

**Figure 1:**
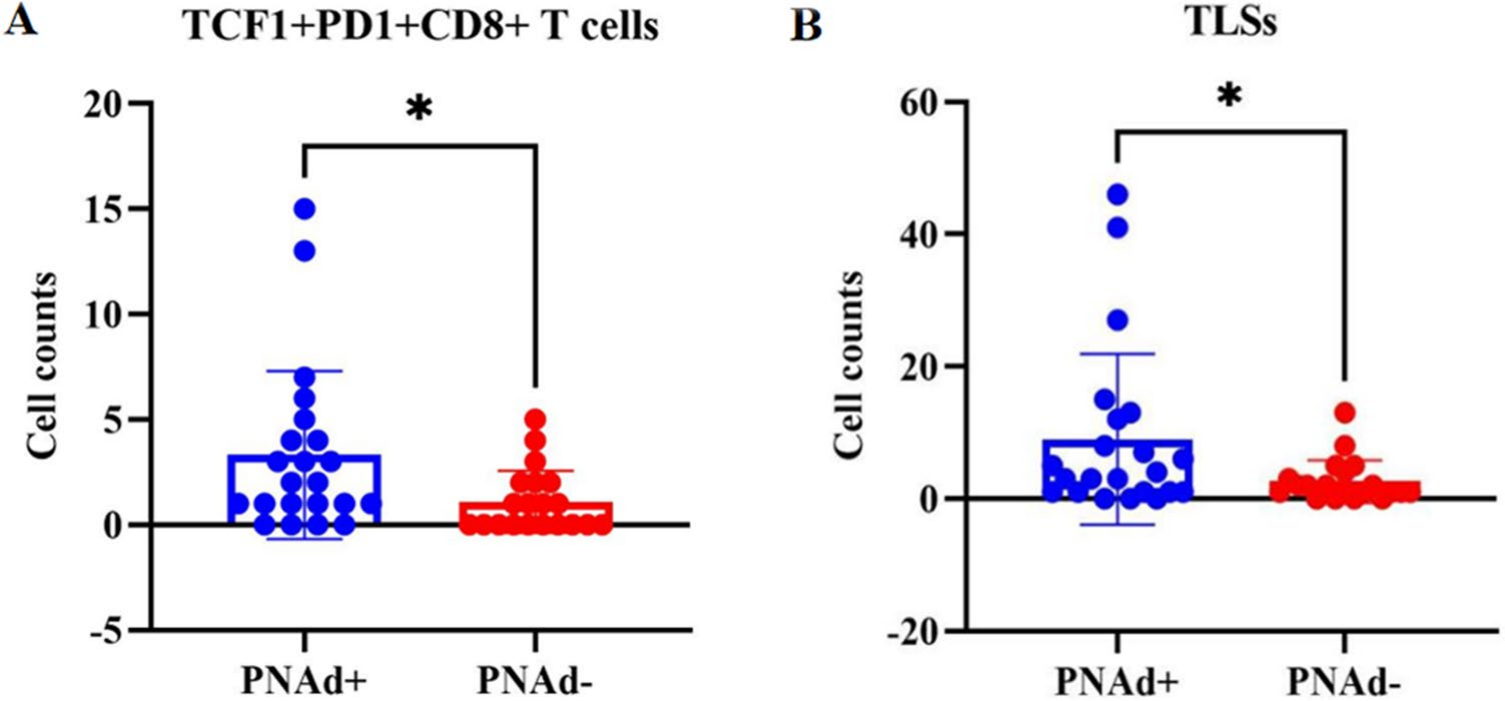
Correlation between HEVs, TLSs and stem-like CD8+ T cells. (**A**) TCF-1+PD-1+CD8+ T cells (**B**) TLSs.

Multiple fluorescence staining of tumor biopsies of 42 patients with advanced NSCLC before treatment reveals that the total number of TLSs (P = 0.0340) and TCF-1+PD-1+CD8+ T cells (P = 0.0221) in tumor microenvironment of PNAd+ patients were higher than those of PNAd- patients (Fig. 1).

Recent studies have revealed the presence of high numbers of stem-like CD8+ T cells and TLSs in the TME is associated with clinical efficacy of immune checkpoint inhibitors (ICIs)^3,19^. Anti-angiogenesis therapy promote the formation of TA-HEVs^3,19^. The above suggested that combined anti-angiogenic and anti-PD-1 therapy might ameliorate ICIs efficacy. Then we retrospective collected real-world data of immunotherapy combined anti-angiogenesis therapy in NSCLC.

### Efficacy of combination therapy in the real world

#### Patients and characteristics

A total of 190 advanced NSCLC patients were enrolled in this real-world retrospective clinical data. The median follow-up time was 12.7 months. Eligible patients were divided into two groups: ICIs combined with anti-angiogenic drugs group (n = 96) and ICIs without anti-angiogenic drugs group (n = 94). Among the combined treatment group, 52 patients received ICIs combined with anti-angiogenesis, 44 patients received ICIs combined with anti-angiogenesis therapy and chemotherapy. A total of 91 patients received ICIs combined with chemotherapy and 3 patients received immunotherapy monotherapy in another group. All of the ICIs were PD-1 inhibitors. The anti-angiogenic drugs are bevacizumab and anlotinib. The chemotherapy regimen was pemetrexed alone, albumin-paclitaxel alone or combined with platinum, and paclitaxel combined with platinum. The included advanced NSCLC patients were mainly male (74.7%), younger than 65 years (51.6%), adenocarcinoma (63.2%), and performance status (PS) score was 0–1 (88.4%). In addition, chi-square test showed that there were no statistically significant differences between the two groups in gender, age, pathological type, EGFR mutation status and other features (Table 1).

**Table 1:** Characteristics of patients (n, %).

Characteristics	Total	Combined with anti-angiogenosis group	Non-combined with anti-angiogenosis group	χ^2^	P
Total	190	96	94		
Sex				0.566	0.452
Male	142 (74.7)	74 (77.1)	68 (72.3)		
Female	48 (25.3)	22 (22.9)	26 (27.7)		
Age, years				0.52	0.471
≥ 65	92 (48.4)	44 (45.8)	48 (59.6)		
< 65	98 (51.6)	52 (54.2)	46 (40.4)		
Histology				0.965	0.617
Adenocarcinoma	120 (63.2)	61 (63.5)	59 (62.8)		
Squamous cell carcinomas	42 (22.1)	23 (24.0)	19 (20.2)		
Other	28 (14.7)	12 (12.5)	16 (17.0)		
Disease stage at diagnosis				0.445	0.505
III	29 (15.3)	13 (13.5)	16 (17.0)		
IV	161 (84.7)	83 (86.5)	78 (83.0)		
PS score				0.73	0.393
0–1	168 (88.4)	83 (86.5)	85 (90.4)		
≥ 2	22 (11.6)	13 (13.5)	9 (9.6)		
EGFR mutation status				4.394	0.111
Negative	89 (46.8)	52 (54.2)	37 (39.4)		
Positive	41 (21.6)	19 (19.8)	22 (23.4)		
Unknown	60 (31.6)	25 (26.0)	35 (37.2)		
Line of therapy				0.317	0.573
1	65 (34.2)	31 (32.3)	34 (36.3)		
≥ 2	125 (65.8)	65 (67.7)	60 (63.8)		
PD-L1 TPS				1.23	0.746
≥ 50%	30 (15.8)	13 (13.5)	17 (18.1)		
1–49%	18 (9.5)	10 (10.4)	8 (8.5)		
< 1%	20 (10.5)	9 (9.4)	11 (11.7)		
Unknown	122 (64.2)	64 (66.7)	58 (61.7)		

#### Survival outcomes

**Figure 2:**
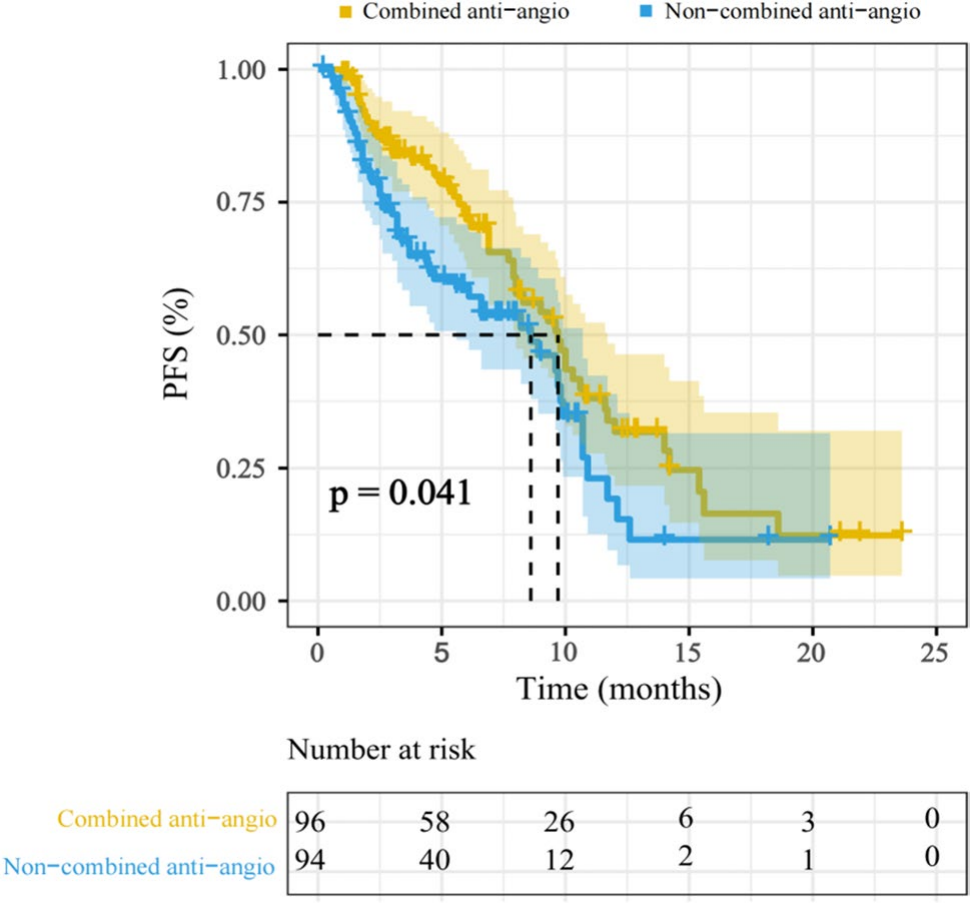
Survival curve of PFS in 190 patients.

The short-term response evaluation showed that the ORR and DCR of the combined anti-angiogenesis therapy group were 39.6% and 82.3%, respectively, which were higher than 25.5% and 75.5% of the another group, but there was only a statistical difference in ORR between the two groups (P = 0.039) (Table 2). Meanwhile, the survival analysis of enrolled patients showed that the median PFS in the combination treatment group was 9.7 months (95% CI: 8.070–11.330) and that in the non-combined with anti-angiogenosis group was 8.6 months (95% CI: 5.522–11.678), the difference was statistically significant (P = 0.041) (Fig. 2).

**Table 2:** The treatment response in the combined with anti-angiogenosis group.

Characteristics	ORR		DCR	
	n (%)	P	n (%)	P
Sex		0.326		0.987
Male	25 (33.8)		65 (87.8)	
Female	6 (27.3)		20 (90.9)	
Age, years		0.597		0.979
≥ 65	13 (29.5)		39 (88.6)	
< 65	18 (34.6)		46 (88.5)	
Histology		0.976		0.115
Adenocarcinoma	20 (32.8)		57 (93.4)	
Squamous cell carcinomas	7 (30.4)		18 (78.3)	
Other	4 (33.3)		10 (83.3)	
Disease stage at diagnosis		0.847		0.354
III	5 (38.5)		13(100.0)	
IV	26 (31.3)		72 (86.7)	
PS score		0.279		**< 0.0001**
0–1	29 (34.9)		82 (98.8)	
≥ 2	2 (15.4)		3 (23.1)	
EGFR mutation status		0.1		0.39
Negative	21 (40.4)		44 (84.6)	
Positive	6 (31.6)		17(89.5)	
Unknown	4 (16.0)		24 (96.0)	
Line of therapy		**0.001**		0.471
1	17 (54.8)		29 (93.5)	
≥ 2	14 (21.5)		56 (86.2)	
PD-L1 TPS		0.131		0.314
≥ 50%	8 (61.5)		13 (100.0)	
1–49%	3 (30.0)		10 (100.0)	
< 1%	2 (22.2)		8 (88.9)	
Unknown	18 (28.1)		54 (84.4)	

Significant values are in bold.

#### Safety analysis

As shown in Table 3, the AEs of ICIs combined with anti-angiogenic drugs were controllable. The most common AEs were thrombocytopenia (38.5%) and fatigue (45.8%), while nausea and vomiting (6.2%) and immune pneumonia (7.3%) were relatively rare. The bleeding was mainly caused by blood in sputum or gingival bleeding, without visceral bleeding or hemorrhagic shock. Moreover, the incidence of grade 3–4 severe AEs was 9.3%, including thrombocytopenia (5.2%), diarrhea or constipation (2.1%), rash (1.0%), and pneumonia (1.0%). No treatment-related AEs resulting in death or withdrawal were found. Thus, immunotherapy combined with anti-angiogenesis therapy is tolerable in the real world.

**Table 3:** Safety analysis in the combined with anti-angiogenosis group.

AEs	Grade 1–2	Grade 3–4
Leukopenia	23 (24.0)	–
Thrombocytopenia	37 (38.5)	5 (5.2)
Nausea and vomiting	6 (6.2)	–
Diarrhea or constipation	11 (11.5)	2 (2.1)
Fatigue	44 (45.8)	–
Hypertension	13 (13.5)	–
Bleeding	9 (9.4)	–
Rash	8 (8.3)	1(1.0)
Hand-foot syndrome	12 (12.5)	–
Pneumonia	7 (7.3)	1 (1.0)

### Correlations between HEVs and clinical efficacy

Pathological sections of 14 patients with advanced NSCLC before treatment were included. These patients were treated with PD-1 inhibitors combined with anti-angiogenesis therapy. The sections were divided into effective group (PFS > 6 months, n = 8) and void group (PFS ≤ 6 months, n = 6) according to PFS.

**Figure 3:**
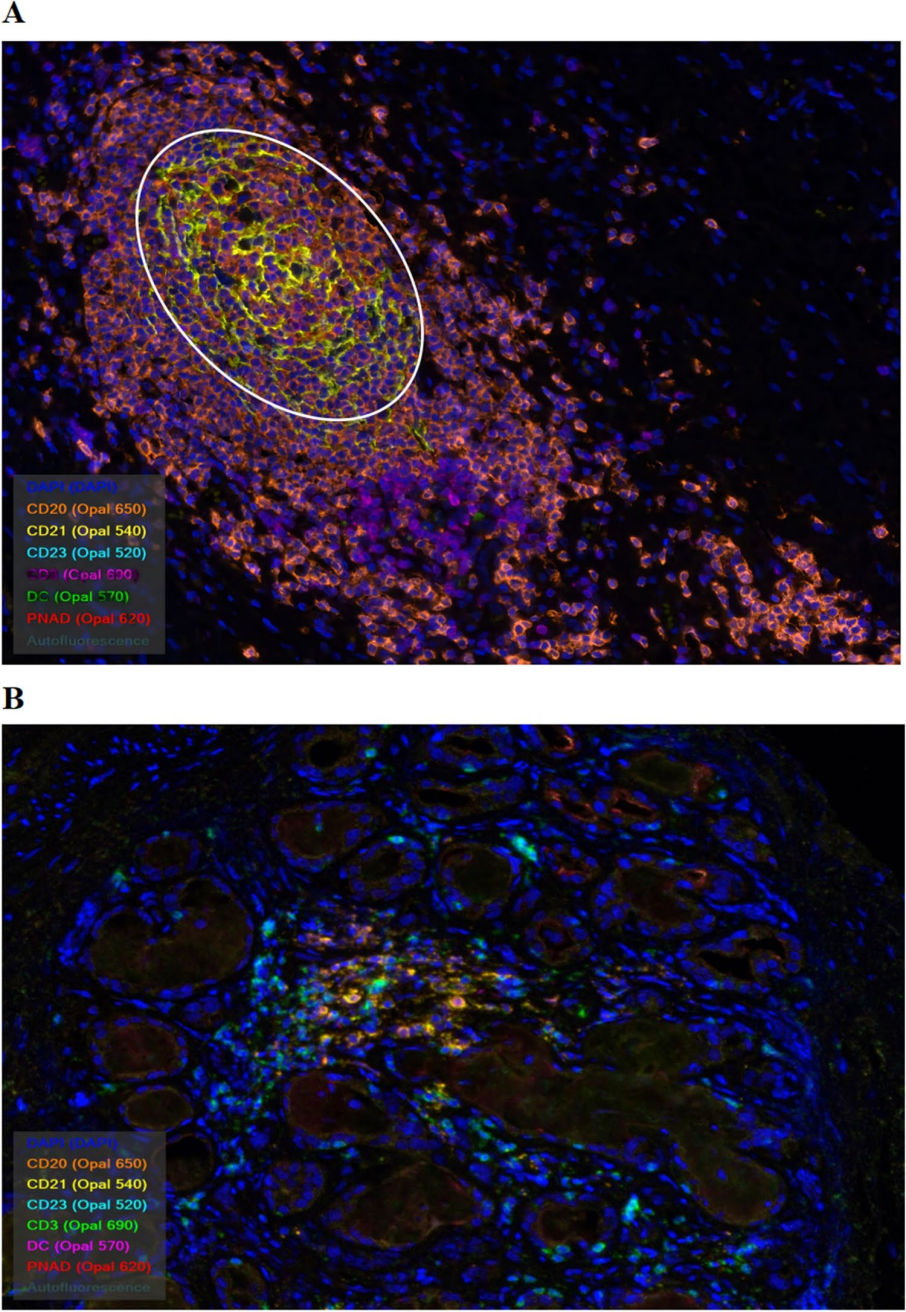
Differences in immune microenvironment components between the two groups. (**A**) The effective group (**B**) the void group.

**Figure 4:**
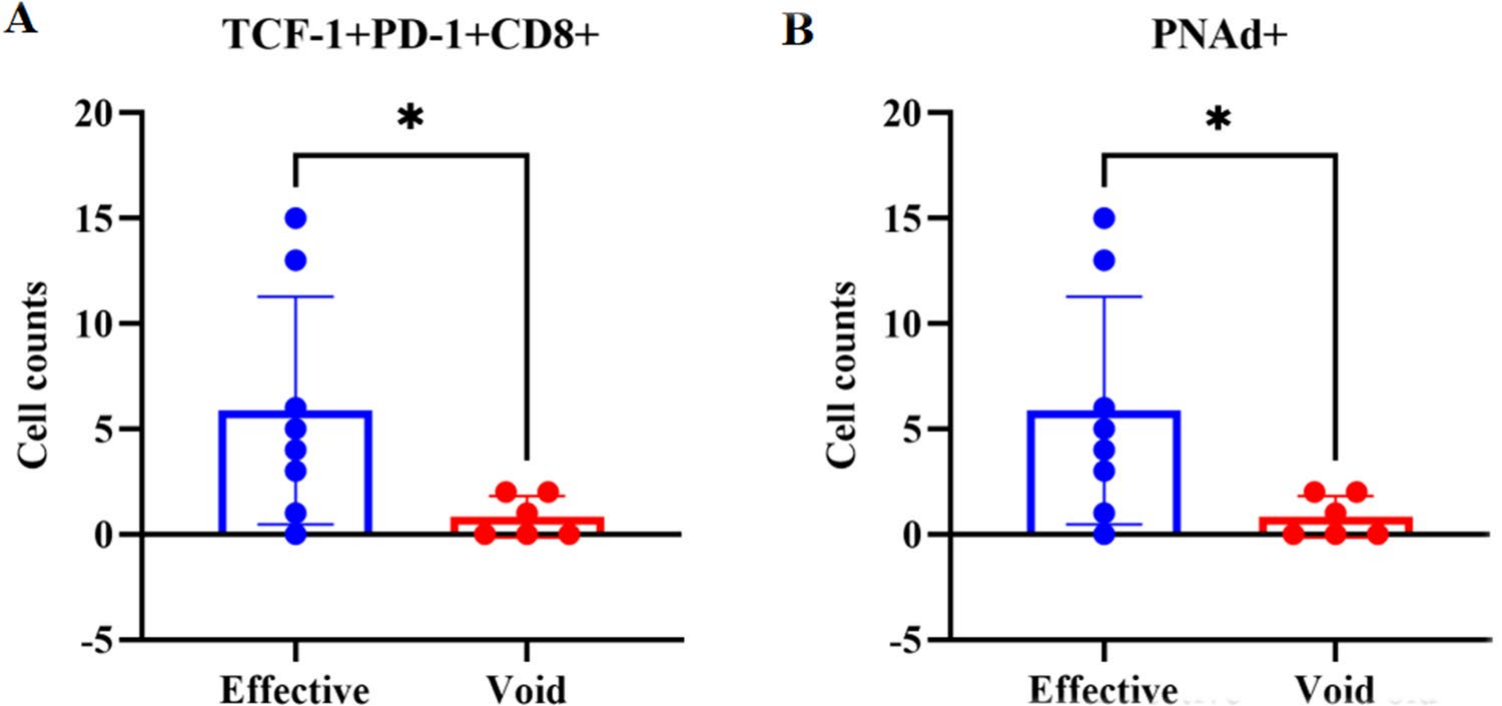
Correlations between HEVs and clinical efficacy.

Multiple immunofluorescence staining showed that PNAd, CD3, CD20, CD21 and CD23 were abundant in the TME of patients in the effective group, CD3/CD20/CD21/CD23 co-localized in the tertiary lymphoid structures, the white circle in Fig. 3A shows a mature tertiary lymphatic structure (Fig. 3). In addition, quantitative analysis found that high PNAd+ (P = 0.0274) and TCF-1+PD-1+CD8+ (P = 0.0421) were positively associated with better efficacy (Fig. 4).

**Figure 5:**
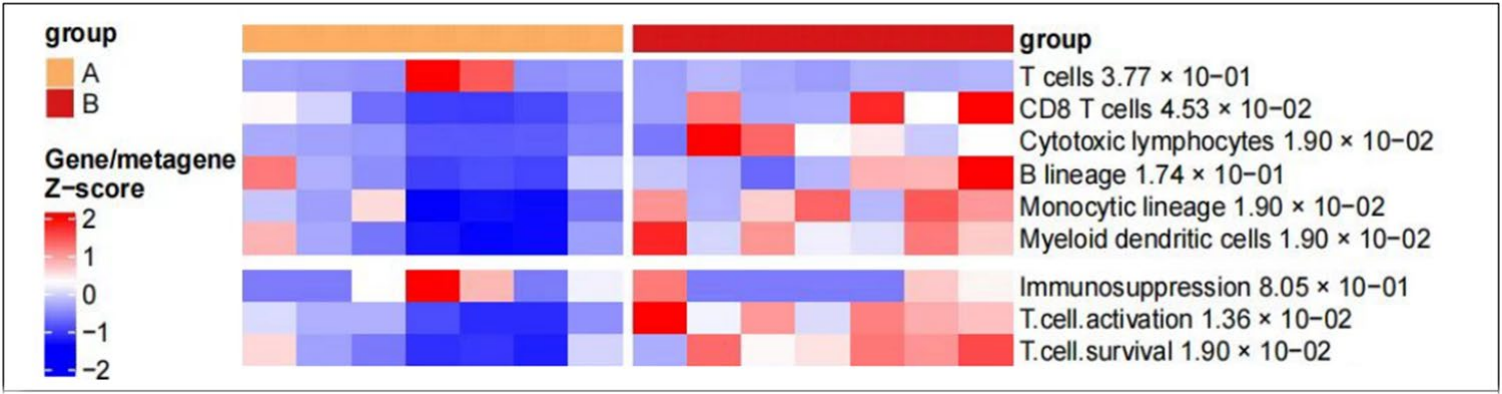
Differences in TME at the genetic level between the two groups (**A**) The void group (**B**) the effective group genetic level.

Furthermore, we collected peripheral blood for RNA sequencing and found that CD8+ T cell, cytotoxic lymphocytes, monocytic lineage, myeloid dendritic cells, T cell activation-related and T cell survival gene expression scores were higher in the effective group (Fig. 5).

## Discussion

Immunotherapy have become a new standard treatment for advanced NSCLC patients due to the enduring anti-tumor effect and survival advantage over chemotherapy^20,21^. However, immunotherapy monotherapy fails to meet public expectations for long-term survival^22^. In order to maximize the effect of immunotherapy, more knowledge about the differential effects and mechanisms of action of combined ICIs versus ICIs monotherapy is of paramount importance.

Trafficking of lymphocytes to tumors is critical for cancer immunity and ICIs immunotherapy ^23–25^. The presence of high numbers of CD8+ T cells in the tumor microenvironment is associated with clinical response to ICIs in NSCLC^6,19^. Recent studies have linked the response to ICIs to TCF-1+PD-1+ stem-like rather than terminally exhausted CD8+ T cells^6,26,27^. Therefore, understanding the mechanisms governing lymphocyte recruitment into tumors at baseline and during ICIs therapy is crucial. HEVs are specialized blood vessels for lymphocyte recruitment in lymph nodes and other lymphoid organs^6,26,27^. TA-HEVs express high levels of sulfated sialomucins recognized by the lymphocyte homing receptor L-selectin (CD62L) and the HEV-specific antibody MECA-79^1,28,29^. MECA-79+ (PNAd+) HEV-like blood vessels are frequently observed in human solid tumors^4,30^. TA-HEVs are present in tertiary lymphoid structures but are most often found in areas containing high densities of T cells and mature dendritic cells and in the absence of B cell-rich TLSs^28,31–33^. In addition, our study suggest that the frequency of TA-HEVs is positively correlated with tumor-infiltrating stem-like CD8+ T cells (TCF-1+PD-1+) in the TME of advanced NSCLC patients (P = 0.0221). All of the above provide a theoretical basis for immunotherapy combined with anti-angiogenesis therapy.

To further explore the safety and efficacy of ICIs combined with anti-angiogenic drugs in the treatment of advanced NSCLC patients in the real world, we conducted a retrospective study. Our results showed that ICIs combined with anti-angiogenic drugs could not only prolong patient’s PFS (9.7 vs 8.6 months), but also had better safety. The most common adverse events were thrombocytopenia (38.5%) and fatigue (45.8%), Nausea and vomiting (6.2%) and immune pneumonia (7.3%) were relatively rare. No treatment-related withdrawal or death occurred. Then, multiple immunofluorescence staining of tumor biopsies before treatment from 14 patients with advanced NSCLC reveals that high endothelial venules (PNAd +) are predictive of better response and survival upon PD-1 inhibitors combined with anti-angiogenesis therapy (P = 0.0274). Moreover, we found at the genetic level that CD8+ T cell, cytotoxic lymphocytes, monocytic lineage, myeloid dendritic cells, T cell activation-related and T cell survival gene expression scores were higher in the effective group.

This study has some limitations. Firstly, due to the strict quality requirements of multiplex immunofluorescence, and the concerning about the overall immune microenvironment of tumors including TLSs, the total sample size and subgroup size were limited. Most of the pathological specimens are small specimens obtained by puncture, and more than 90% of the specimens do not meet the research requirements. Therefore, among the 96 NSCLC patients receiving combination of anti-angiogenic and anti-PD-1 therapy in Wuhan University People's Hospital from January 2018 to December 2021, only 14 specimens met the study requirements. Then, there may be some heterogeneity in the chemotherapy regimen of enrolled patients. In addition, patients were followed up for a short period of time and OS was not yet available for analysis. In view of the above limitations, our conclusions may need to be further confirmed with a larger sample size, which is also our future focus and research direction.

## Conclusion

In NSCLC, the density of TA-HEVs is significantly associated with TLSs and stem-like CD8+ T cells, suggesting that immunotherapy combined with anti-angiogenesis therapy is a feasible treatment option. We find that immunotherapy combined with anti-angiogenesis therapy has good efficacy and safety in the real world for advanced NSCLC patients. In addition, TA-HEVs number is positively correlated with the efficacy of combination therapy. However, its sensitivity and specificity need to be further explored in more studies with larger sample size.

### Supplementary Information

Supplementary Figure 1.

## Data Availability

The date that support the findings of this study are available on request from the corresponding author. The data are not publicly available due to privacy or ethical restrictions. All data generated or analyzed during this study are included in this published article.
