# Supplementary figures and images for: High endothelial venules predict response to PD-1 inhibitors combined with anti-angiogenesis therapy in NSCLC
Source: Sci Rep. 2023 Sep 30;13:16468. doi: 10.1038/s41598-023-43122-w (PMC10543372; doi:10.1038/s41598-023-43122-w)

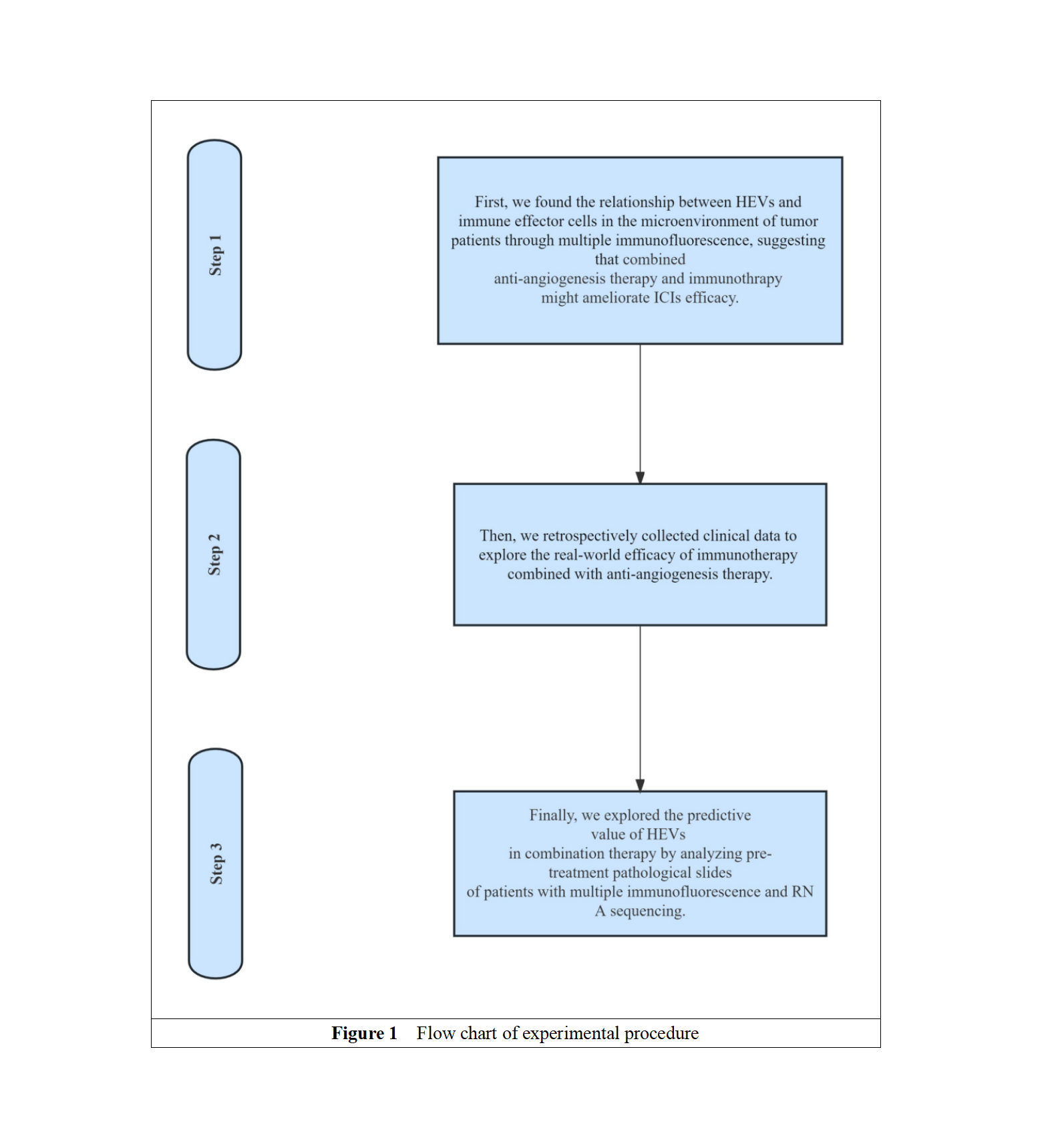

Supplement: Supplementary file 1 — Supplementary Figure 1. [file 41598_2023_43122_MOESM1_ESM.tif]
